# Blocked Two-Level Regular Designs with Individual Aliased Effect Number Pattern

**DOI:** 10.3390/e28030340

**Published:** 2026-03-18

**Authors:** Min Han, Shengli Zhao, Tao Sun

**Affiliations:** School of Statistics and Data Science, Qufu Normal University, Qufu 273165, China; hanmin@qfnu.edu.cn (M.H.);

**Keywords:** blocked designs, blocked individual aliased effect number pattern, minimum aberration, 62K05, 62K15

## Abstract

This paper proposes a blocked individual aliased effect number pattern (BI-AENP) for regular blocked designs and establishes its relationships with the core patterns of several existing optimality criteria. We develop an algorithm to compute the BI-AENP. A catalogue of 16-, 32-, and 64-run BI-AENP $2^{n-k}:2^{r}$ designs is presented, together with comparisons with the minimum aberration and clear effects criteria.

## 1. Introduction

The core challenge of blocked designs lies in how to effectively separate the factor (treatment) effects and block effects within a limited number of experiments. This process is essentially similar to the signal separation problem in information theory, where the factor effects are the target signals, the block effects are structural noise, and the design matrix is equivalent to a specific channel coding scheme. Meanwhile the confounding relationship between treatment effects and block effects defines the noise characteristics of the channel. This information perspective not only provides new indicators for evaluating design efficiency but also establishes a theoretical foundation for exploring the optimal experimental coding strategy under information constraints. The authors of [1] revealed that factorial designs and linear coding are mathematically isomorphic, which lays a theoretical foundation for examining the confounding structure of the design from an information theory perspective. For more information on the connections between information theory, especially coding theory, and experimental designs, see [2].

In blocked designs, effects are assumed to follow the effect hierarchy principle (EHP): (i) lower-order treatment effects are more likely to be important than higher-order ones, (ii) treatment effects of the same order are equally important, (iii) interactions between treatment and block factors are negligible, and (iv) the main effects and interactions of the block factors are equally important. These assumptions have been discussed in [3,4].

Over the past few decades, significant contributions have been made in developing optimality criteria for blocked designs under the EHP. Under the minimum aberration (MA) criterion, refs. [5,6,7,8] proposed four different blocking wordlength patterns and derived optimal blocked designs through sequential minimization of the components of the patterns. Under the clear effects (CE) criterion, ref. [9] introduced the concepts of clear main effects and clear two-factor interactions (2fis) in blocked designs, aiming to maximize the number of clear effects. Refs. [10,11] extended the general minimum lower-order confounding (GMC) criterion to the blocked designs and proposed the B-GMC and $B^{1}$-GMC criteria, respectively.

Different criteria address effect aliasing from distinct perspectives. The MA criterion is more suitable when all factors are equally important. In contrast, the CE and GMC criteria can incorporate prior information on factor importance, but they primarily focus on reducing overall confounding of the lower-order effects. However, existing criteria cannot quantify the aliasing degree of a specific effect with others. Therefore, ref. [12] proposed the individual aliased effect number pattern (I-AENP) for two-level designs and established its relationship with the core patterns of existing criteria. Motivated by the above discussion, we conduct a systematic study on the I-AENP in the context of blocked designs.

The remainder of this paper is organized as follows. Section 2 introduces the blocked individual aliased effect number pattern (BI-AENP) for blocked designs. Section 3 establishes the relationships between the BI-AENP and the characteristic patterns of the MA, CE and GMC criteria. Section 4 presents an algorithm for computing the BI-AENP and demonstrates its applications through an illustrative example. Section 5 summarizes this paper and discusses future research directions. Some 16-, 32-, and 64-run BI-AENP blocked designs are tabulated in the Supplementary Materials.

## 2. Preliminaries and the BI-AENP

Let q=n−k and $N=2^{q}$. Define the N×(N−1) matrix

$$H_{q}=\left\{1,2,12,3,13,23,123,\ldots ,123\ldots q\right\}$$

with columns in Yates order, where $1={\left(-1,1,\ldots ,-1,1\right)}^{\prime }$, 2=(−1,−1,1,1,…,−1,${-1,1,1)}^{\prime },\ldots ,$ and $q={\left(-1,\ldots ,-1,1,\ldots ,1\right)}^{\prime }$ stand for its *q* independent columns. The other columns are obtained as Hadamard products of the *q* independent columns, say $12={\left(1,-1,-1,1,\ldots ,1,-1,-1,1\right)}^{\prime }$.

Let $D=(D_{t}:D_{b})$ denote a regular blocked $2^{n-k}:2^{r}$ design taken from $H_{q}$, where $D_{t}$ is an N×n matrix with each column representing a treatment factor and $D_{b}$ is an $N\times (2^{r}-1)$ matrix corresponding to the block factors and their interactions. Such a blocked design is determined by a defining relation or defining contrast subgroup, denoted by *G*, which is generated by *k* independent treatment defining words and *r* independent block defining words. Let $A_{i,0}$ be the number of treatment defining words of length *i* and $A_{i,1}$ be the number of *i*th-order treatment effects (*i*-factor interactions, *i*fis) aliased with block effects. Throughout this paper, we focus on blocked designs in which no treatment main effect is aliased with other treatment main effects or with block effects. In other words, we assume that $A_{1,0}=A_{2,0}=A_{1,1}=0$. The vectors $W_{t}=\left(A_{3,0},\ldots ,A_{n,0}\right)$ and $W_{b}=\left(A_{2,1},\ldots ,A_{n,1}\right)$ are referred to as the treatment and block wordlength patterns, respectively. Refs. [6,8] independently proposed the same blocked minimum aberration criterion, which sequentially minimizes

$$\begin{array}{c} (A_{3,0},A_{2,1},A_{4,0},\ldots ,A_{2j-1,0},A_{2j,1},A_{2j,0},\ldots ). \end{array}$$

Suppose $A_{l,j}\left(D\right)$ is the first nonzero element in the above sequence; then the resolution of *D* is *l* if j=0 and $2l^{-}$ if j=1 [8]. For more details on regular $2^{n-k}:2^{r}$ designs, see [2].

**Example 1.**  *Consider a $2^{6-2}:2^{2}$ design D = (1, 2, 3, 4, 123, 124: 134, 234, 12) taken from $H_{4}$. For an experiment with six factors and sixteen runs that must be blocked into four blocks, the experimenter may assign the treatment factors to the six columns 1,2,3,4,123,124 and allocate the sixteen runs into four blocks using the columns* 134 *and* 234 *as two independent block factors. Then the corresponding defining contrast subgroup G is*

$$G\;=\;\{1235,1246,3456,134b_{1},234b_{2},245b_{1},145b_{2},236b_{1},136b_{2},\;12b_{1}b_{2},156b_{1},256b_{2},35b_{1}b_{2},46b_{1}b_{2},123456b_{1}b_{2}\}.$$

*The corresponding treatment and block wordlength patterns are*

$$W_{t}=\left(0,3,0,0\right)\;\mathrm{and}\;W_{b}=\left(3,8,0,0,1\right),$$

*respectively. Since $A_{3,0}=0$ and $A_{2,1}=3\neq 0$, the resolution R is ${\mathrm{IV}}^{-}$.*

A regular $2^{n-k}:2^{r}$ design has $2^{n}-1$ treatment effects. These effects are partitioned into four classes: the *g*-, *b*-, *m*- and ϕ-classes, which respectively consist of the grand mean, block effects, main treatment effects, and the remaining interaction effects. Let δijl∗ be the number of *j*th-order treatment effects aliased with the *l*-th ith-order treatment effect in the ∗-class, ∗=g,b,m,ϕ, i,j=0,1,…,n, l=1,…,Li∗, where Li∗ is the number of ith-order treatment effects in the ∗-class, ∑∗=g,b,m,ϕLi∗=Li and Li=ni. The set

(1) FB=Fijg,Fijb,Fijm,Fijϕ

is called a blocked individual aliased effect number pattern (BI-AENP) of *D*, where Fij∗=(Fij1∗,…,FijLi∗∗) is the sequence with elements δijl∗ arranged in nondecreasing order. Although (1) may seem complicated, our main concern remains the confounding between lower-order effects.

For blocked designs with resolution R≥III, assuming that the third- and higher-order effects of treatment factors are negligible, this paper is mainly concerned with the confounding between the main effects and 2fis. Thus the BI-AENP can be simplified as

(2) FB=F12∗,F21∗,F22∗,∗=b,m,ϕ.

Note that Fijg(i,j=1,2) does not appear in (2), as the *g*-class contains neither main effects nor 2fis.

According to the EHP, main effects are prioritized. For the vector F12m=(F121m,…,F12nm), which characterizes the confounding of main effects with 2fis, the components of F12m should be sequentially minimized. For F21∗,∗=b,m,ϕ, noting that both F21b and F21ϕ are empty sets, we only need to consider F21m. In addition there are ∑l=1nF12lm 2fis aliased with the main effects; the remaining ν=L2−∑l=1nF12lm 2fis are not aliased with any main effect. As a binary vector, the first ν entries of F21m are 0 s and the last ∑l=1nF12lm entries are 1 s. Thus, F21m can be dropped because it is determined by the preceding term F12m. Furthermore, note that

ν=L2−∑l=1nF12lm=∑k=0L2ϕ∑l=1L2ϕI(F22lϕ=k)+∑k=0L2b∑l=1L2bI(F22lb=k),

Hereafter, I(·) denotes the indicator function. This equality indicates a relationship between the vectors F22b and F22ϕ. Accordingly, the subsequent analysis will focus on the relative ordering among the vectors F22b, F22m and F22ϕ. Among the three classes, the 2fis in the ϕ-class have the lowest degree of aliasing, as they are not confounded with any block effects or main treatment effects. Since the 2fis in the *b*-class are aliased with block effects, the vector F22b can be dropped. As a result, the BI-AENP can be reduced to the ordered vector (F12m;F22ϕ;F22m),

or

(3) F121m,F122m,…,F12nm;F221ϕ,F222ϕ,…,F22L2ϕϕ;F221m,F222m,…,F22L2mm.

We also refer to (3) as the BI-AENP. Clearly, a $2^{n-k}:2^{r}$ design that sequentially minimizes (3) can minimize the confounding between the treatment main effects and 2fis. Such a blocked design is called the BI-AENP design.

**Example 2.**  *Consider a $2^{5-1}:2^{3}$ design $D_{1}$ determined by the defining relationship*

$$\begin{array}{cc} I & =1345=14b_{1}=23b_{2}=13b_{3}=35b_{1}=1245b_{2}=45b_{3}=1234b_{1}b_{2}=34b_{1}b_{3}\\ & =12b_{2}b_{3}=24b_{1}b_{2}b_{3}=25b_{1}b_{2}=15b_{1}b_{3}=2345b_{2}b_{3}=1235b_{1}b_{2}b_{3}. \end{array}$$

*By a simple partitioning, all effects can be classified into four distinct classes:*

$$\begin{array}{cc} & \mathrm{g-class}:\{I,1345\},\\ & \mathrm{b-class}:\{14,35\left(=b_{1}\right);23,1245\left(=b_{2}\right);13,45\left(=b_{3}\right);25,1234\left(=b_{1}b_{2}\right);\\ & \;\;\;\;15,34\left(=b_{1}b_{3}\right);12,2345\left(=b_{2}b_{3}\right);24,1235\left(=b_{1}b_{2}b_{3}\right)\},\\ & \mathrm{m-class}:\{1,345;2,12345;3,145;4,135;5,134\},\\ & \mathrm{\phi -class}:\{123,245;124,235;125,234\}. \end{array}$$

*In the m-class, since no 2fi is aliased with the main treatment effects, it follows that δ121m=δ122m=δ123m=δ124m=δ125m=0. Hence, F12m=(0,0,0,0,0). Moreover, since there are no 2fis in the m-class and ϕ-class, F22ϕ and F22m do not exist.*

## 3. Relationships with the Other Characteristic Patterns

In this section, we establish the relationships between the BI-AENP and the characteristic patterns of the MA, CE and GMC criteria. We first focus on its relationship with the wordlength pattern (WLP).

For a $2^{n-k}:2^{r}$ design $D=(D_{t}:D_{b})$ of resolution R≥III, $D_{t}$ can be regarded as an unblocked $2^{n-k}$ design. For $D_{t}$, let $\delta_{ij}^{l}\left(D_{t}\right)$ be the number of *j*th-order effects aliased with the *l*-th *i*th-order effect, i,j=0,1,…,n, $l=1,\ldots ,L_{i}$. Let

$$F_{ij}\left(D_{t}\right)=\left(F_{ij}^{1}\left(D_{t}\right),F_{ij}^{2}\left(D_{t}\right),\ldots ,F_{ij}^{L_{i}}\left(D_{t}\right)\right)$$

be the sequence with elements $\delta_{ij}^{l}\left(D_{t}\right),l=1,\ldots ,L_{i}$, arranged in nondecreasing order. Then, $F_{ij}\left(D_{t}\right)$ is referred to as the I-AENP of $D_{t}$. From the definition of F1jm(D), we have F1jm(D)=F1j(Dt). Therefore, $A_{i,0}$ can be directly derived from Theorem 1 in [12]. Theorem 1 further develops these results and shows that both $W_{t}$ and $W_{b}$ can be expressed as functions of the BI-AENP.

**Theorem 1.**  *For a $2^{n-k}:2^{r}$ design $D=(D_{t}:D_{b})$ of resolution R≥III, we have*

Ai,0=F0ig, i=3,…,n,Aj,1=∑p=0Ljb−1∑l=1LjbI(Fjjlb=p), j=2,…,n.

**Proof.**  Since $A_{i,0}$ represents the number of *i*fis of treatment factors in the *g*-class, it is obvious that the first equality holds.

Consider the aliasing frequency Fjjlb of the *j*fis in the *l*-th alias set of *b*-class, l=1,2,…,Ljb. For a given *p*, ∑l=1LjbI(Fjjlb=p) equals the number of *j*fis aliased with *pj*fis in the alias sets of the *b*-class. Then, ∑p=0Ljb−1∑l=1LjbI(Fjjlb=p) is the total number of *j*fis aliased with the block effects, which is $A_{j,1}$. The second equality follows.    □

Next, we examine the relationship between the BI-AENP and the CE. It is generally assumed that the third- and higher-order interactions of treatment factors are negligible. Furthermore, according to the EHP of blocked designs, interactions between treatment and block factors are negligible, and estimation of block effects is typically not of interest. Therefore, we focus on the aliasing relationships of the main treatment effects and 2fis. A main treatment effect or 2fi is said to be clear if it is not aliased with any main treatment effects or 2fis, nor with any block effects. Let $C_{1}$ and $C_{2}$ be the numbers of clear main effects and 2fis, respectively. From the definition of Fij∗, the following result can be obtained.

**Theorem 2.**  *For a $2^{n-k}:2^{r}$ design of resolution R≥III, we have*

C1=∑l=1nI(mF12l=0) and C2=∑l=1L2ϕI(ϕF22l=0).

**Proof.**  Recall that all treatment effects are classified into four classes with all main effects belonging to the *m*-class. In particular, the number of clear main effects is equal to the number of main effects in the *m*-class that are not aliased with any 2fis. That is $C_{1}=\sum_{l=1}^{n}I{(}^{m}F_{12}^{l}=0)$.

We consider blocked designs with resolution R≥III. In such designs, there is no 2fi in the *g*-class, and all 2fis are distributed among the *m*-, *b*-, and ϕ-classes. The 2fis in the *m*-class and the *b*-class are not clear, as they are aliased with either treatment main effects or block effects. Therefore, according to the definition of clear 2fi, the number of clear 2fis is equal to the number of 2fis in the ϕ-class that are not aliased with any other 2fis, which is ∑l=1L2ϕI(ϕF22l=0).    □

Finally, we consider the relationship between the BI-AENP and the AENP. For a $2^{n-k}:2^{r}$ design *D*, let Cj(p)i#∗ denote the number of *i*fis aliased with *p j*fis in the ∗-class, ∗=g,b,m,ϕ. Thus, we obtain the set

(4) C#B={Cji#g,Cji#b,Cji#m,Cji#ϕ,i,j=0,1,…,n},

where

(5) C00#∗=(0),Cji#∗=(Cj(p)i#∗,k=0,1,…,Lj∗),fori,j≠0,L0=L1=1,∑∗=g,b,m,ϕLj∗=Lj=nj,j=2,…,n,and∗=g,b,m,ϕ.

We call the set (4) with (5) a blocked aliased effect number pattern (B-AENP). The design obtained by sequentially maximizing the B-AENP is called a $B^{1}$-GMC design. Theorem 3 follows directly from the definitions of Cj(k)i#∗ and Fij∗.

**Theorem 3.**  *For a $2^{n-k}:2^{r}$ design D, the B-AENP can be expressed as a function of FB as follows:*

Cj(p)i#∗=∑l=1Li∗I(Fijl∗=p),i,j=1,…,n,k=0,1,…,Lj∗.

**Proof.**  For a given *p*, ∑l=1Li∗I(Fijl∗=p) is the total number of *i*fis aliased with *p j*fis in the alias sets of the ∗-class. According to the definitions of Cj(p)i#∗, this equality follows directly.    □

## 4. Algorithm for BI-AENP

In this section, we present an algorithm for computing the BI-AENP. Before describing the algorithm in detail, we note that (3) can be further simplified, as established in Theorem 4. This simplification forms the basis for the computational procedure.

**Theorem 4.**  *For a BI-AENP $2^{n-k}:2^{r}$ design, if $n\leq 2^{n-k-1}$, then the resolution of the corresponding $2^{n-m}$ design is at least IV.*

**Proof.**  According to Theorem 1 in [9], if $n\leq 2^{n-k-1}$, there exist $2^{n-k}:2^{r}$ designs such that all the treatment main effects are clear. Let $D=(D_{t}:D_{b})$ be the optimal design under the BI-AENP. It follows that all the treatment main effects of $D=(D_{t}:D_{b})$ are clear, and hence all the main effects of the corresponding $2^{n-k}$ design are also clear. Consequently, the corresponding $2^{n-k}$ design must have resolution at least IV.    □

   According to Theorem 4, when $n\leq 2^{n-k-1}$, F22lm=0 for a BI-AENP $2^{n-k}:2^{r}$ design, $l=1,\ldots {,}^{m}L_{2}$. Thus (3) can be further simplified to

F121m,F122m,…,F12nm;F221ϕ,F222ϕ,…,F22L2ϕϕ.

Two blocked designs are said to be isomorphic if the defining contrast subgroup of one can be obtained from that of the other one by relabeling the treatment factors and block factors, respectively. In ranking and selecting designs, isomorphic designs should be regarded as equivalent [2]. For a $2^{n-k}:2^{r}$ design *D* taking from $H_{q}$, up to isomorphism, let the independent columns of *D* be 1,…,q, where q=n−k. Then each of the columns of $H_{q}$, which are arranged in Yates order and ranked from 1 to N−1, corresponds to an aliasing relation of *D*. Note that the defining relation corresponds to the identity column *I*, which is ranked 0 (Algorithm 1).
**Algorithm 1** Calculate BI-AENP for $2^{n-k}:2^{r}$ Designs*Input:*  Defining relation of the $2^{n-k}:2^{r}$ design *D*.*Step 1.* Generate the aliasing relations of *D*. Here, all interaction terms involving both treatment and block factors are omitted.*Step 2.* Calculate the aliasing relation matrix C=(A B), where $A=(a_{uv})$, $a_{uv}$ is the number of *v*fis of the treatment factors in the *u*th aliasing relation, u=0,1,…,N−1, v=1,2; $B=(b_{uw})$, $b_{uw}$ is the number of *w*fis of the block factors in the *u*th aliasing relation, w=1,…,r.*Step 3.* Calculate BI-AENP from the aliasing relation matrix *C*.
For $a_{u1}\neq 0$, select the pairs $\left(a_{u1},a_{u2}\right)$, u=0,1,…,N−1, and let F12m be the vector with components $a_{u2}$ arranged in nondecreasing order and each $a_{u2}$ repeating $a_{u1}$ times.For $a_{u1}=0$ and $b_{uw}=0$, select the components $a_{u2}>0$, u=0,1,…,N−1, w=1,…,r, let F22ϕ be the vector with components $a_{u2}-1$ arranged in nondecreasing order and each $a_{u2}-1$ repeating $a_{u2}$ times.


The algorithm can be extended to the more general form of (1), thereby achieving broader applicability. Example 3 provides a detailed illustration of the computational procedure, thereby facilitating a deeper understanding of the algorithm.

**Example 3.**  *Consider a $2^{5-2}:2^{1}$ design $D_{2}$ determined by the following defining relation:*

$$\begin{array}{c} I=124=135=2345=23b=134b=125b=45b. \end{array}$$

*To save space, we omitted all the terms of interactions containing both treatment and block factors. Thus, its aliasing relations are as follows:*

$$\begin{array}{cc} & 1=24=35=12345,\\ & 2=14=1235=345,\\ & 12=4=235=1345,\\ & 3=1234=15=245,\\ & 13=234=5=1245,\\ & 23=134=125=45=b,\\ & 123=34=25=145. \end{array}$$

*The aliasing relation matrix can be obtained by counting the effects in the defining relation and aliasing relations:*

$$C=\left(A\;B\right)=\left(a_{u1}a_{u2}b_{u1}\right)=\left(\begin{array}{ccc} 0 & 0 & 0\\ 1 & 2 & 0\\ 1 & 1 & 0\\ 1 & 1 & 0\\ 1 & 1 & 0\\ 1 & 1 & 0\\ 0 & 2 & 1\\ 0 & 2 & 0 \end{array}\right).$$

*To calculate F12m, we first select the pairs $\left(a_{u1},a_{u2}\right)$ with $a_{u1}\neq 0$, resulting in five pairs:*

$$\begin{array}{cc} & \left\{\left(a_{11},a_{12}\right),\left(a_{21},a_{22}\right),\left(a_{31},a_{32}\right),\left(a_{41},a_{42}\right),\left(a_{51},a_{52}\right)\right\}\\ = & \{(1,2),(1,1),(1,1),(1,1),(1,1)\}. \end{array}$$

*By ranking $\left\{a_{12},a_{22},a_{32},a_{42},a_{52}\right\}=\left\{2,1,1,1,1\right\}$ in nondecreasing order, we get F12m=(1,1,1,1,2).*

*To calculate F22ϕ, by choosing the components $a_{u1}=0$, $b_{u1}=0$ and $a_{u2}>0$, we obtain the set $\left\{a_{72}\right\}=\left\{2\right\}$; then $\left\{a_{72}-1\right\}=\left\{1\right\}$. Therefore, F22ϕ=(1,1), where $a_{72}-1=1$ is repeated two times since $a_{72}=2$.*

In the Supplementary Materials, Table S1 provides the numbering scheme for the elements of $H_{6}$, which are represented by consecutive serial numbers 1,2,3,4,5,6,… rather than by the original notation 1,2,12,3,13,23,… Tables S2–S4 present the 16-, 32-, and 64-run BI-AENP $2^{n-k}:2^{r}$ designs, along with the corresponding comparisons with the MA and CE criteria. For each design in Tables S2–S4, only treatment additional columns (Treatment Add.) and block additional columns (Block Add.) are listed, while their independent columns are omitted. The tables include F12m and F12ϕ, representing elements of the BI-AENP. In addition, the WLP $W_{t}$ and $W_{b}$, the numbers of clear main effects ($C_{1}$), and the numbers of clear 2fis ($C_{2}$) are listed.

## 5. Conclusions

Blocking is commonly used in practical applications to reduce systematic variation and increase the precision of effect estimation. When experimenters are interested in certain effects, the confounding information of individual effects becomes particularly important. In this paper, we first introduce the BI-AENP for measuring the aliasing degrees of individual effects. Then, we establish the relationships between the BI-AENP and the characteristic patterns of the MA, CE, and GMC criteria. Lastly, we give an algorithm for calculating the simplified BI-AENP (3). Clearly, the algorithm can be easily generalized to calculate the BI-AENP (1). However, the computational efficiency of the algorithm requires further improvement. Thus, developing efficient algorithms to reduce computational costs is a crucial challenge for future research.

In practical situations, fractional factorial designs with factors at *s* levels, s≥2, are commonly used, especially when investigators anticipate curvature effects of quantitative factors or when qualitative factors have multiple levels. More generally, designs with the factors at different levels, say one factor at $s^{r}$ levels and *n* factors at *s* levels, or one factor at $s^{r_{1}}$ levels, a second factor at $s^{r_{2}}$ levels, and *n* factors at *s* levels, are also to be considered. Therefore, extending the I-AENP to multi-level or mixed-level designs is theoretically feasible. We will investigate this direction in future research.

In addition, blocked two-level designs are often implemented using widely used software packages. For example, JMP 15 provides options to specify blocks and automatically assigns runs to blocks while maintaining the design properties. Exploring the practical implementation of such designs in software like JMP will be considered in our future work, which may further enhance the applicability of these designs in real experimental settings.

## Data Availability

No new data were created or analyzed in this study.

## Supplementary Materials

The following supporting information can be downloaded at https://www.mdpi.com/article/10.3390/e28030340/s1. Table S1: Numbering of the elements of $H_{q}$ for 16-, 32-, and 64- run designs; Table S2: 16-run B-I-AENP $2^{n-k}:2^{r}$ designs and comparisons with the MA and CE criteria; Table S3: 32-run B-I-AENP $2^{n-k}:2^{r}$ designs and comparisons with the MA and CE criteria; Table S4: 64-run B-I-AENP $2^{n-k}:2^{r}$ designs and comparisons with the MA and CE criteria.
