# Supplementary material for: Blocked Two-Level Regular Designs with Individual Aliased Effect Number Pattern
Source: Entropy (Basel). 2026 Mar 18;28(3):340. doi: 10.3390/e28030340 (PMC13025214; doi:10.3390/e28030340)
Supplement: Supplementary file 1 [file entropy-28-00340-s001.zip › entropy-4160597-supplementary.pdf]

Supplementary Materials for "Blocked Two-Level Regular Designs with Individual Aliased Effect Number Pattern"

Min Han, Shengli Zhao, Tao Sun*

School of Statistics and Data Science, Qufu Normal University, Qufu 273165, China

Table S1: Numbering of the elements of H_q for 16-, 32-, and 64- run designs.

Number	**1**	**2**	3	**4**	5	6	7	**8**	9
Element	1	2	12	3	13	23	123	4	14
Number	10	11	12	13	14	15	**16**	17	18
Element	24	124	34	134	234	1234	5	15	25
Number	19	20	21	22	23	24	25	26	27
Element	125	35	135	235	1235	45	145	245	1245
Number	28	29	30	31	**32**	33	34	35	36
Element	345	1345	2345	12345	6	16	26	126	36
Number	37	38	39	40	41	42	43	44	45
Element	136	236	1236	46	146	246	1246	346	1346
Number	46	47	48	49	50	51	52	53	54
Element	2346	12346	56	156	256	1256	356	1356	2356
Number	55	56	57	58	59	60	61	62	63
Element	12356	456	1456	2456	12456	3456	13456	23456	123456

(i) The table gives the serial numbers of the elements of H_6.
(ii) Independent elements are numbered **1**, **2**, **4**, **8**, **16**, **32** in boldface.

*Corresponding author.
Email addresses: hanmin@qfnu.edu.cn (Min Han), suntao911@qfnu.edu.cn (Tao Sun*)

Table S2: 16-run BI-AENP $2^{n-k}:2^r$ designs and comparisons with the MA and CE criteria.

n	k	r	Treatment Add.	Block Add.	BI-AENP ${}^{m}F_{12}$; ${}^{\phi}F_{22}$	WLP $(A_{3,0},\ldots,A_{6,0})$ $(A_{2,1},\ldots,A_{5,1})$	Cs C_1, C_2
4	0	1		15	$0^4; 0^6$	$(0,0,0,0)(0,0,1,0)$	$4,6$
	0	2		3 14	$0^4; 0^5$	$(0,0,0,0)(1,2,0,0)$	$4,5$
	0	3		3 6 12	$0^4; -$	$(0,0,0,0)(6,0,1,0)$	$4,0$
5	1	1	15	14	$0^5; 0^9$	$(0,0,1,0)(1,1,0,0)$	$5,9$
	1	2	15	3 14	$0^5; 0^7$	$(0,0,1,0)(3,3,0,0)$	$5,7$
	1	3	13	3 6 10	$0^5; -$	$(0,1,0,0)(10,0,4,0)$	$5,0$
6	2	1	7 14	11	$0^6; -, 1^{12}, 2^3$	$(0,3,0,0)(0,4,0,0)$	$6,0$
	2	2	7 14	6 11	$0^6; -, 1^{12}$	$(0,3,0,0)(3,8,0,0)$	$6,0$
	2	3	7 14	3 6 9	$0^6; -$	$(0,3,0,0)(15,0,12,0)$	$6,0$

(i) Each design is represented by 1, 2, 4, 8 and the numbers specified under "Treatment Add." and "Block Add.", which denote the treatment additional columns and block additional columns, respectively.
(ii) "a^s" denotes s successive a components.
(iii) "$-$" denotes the absence of an element.
(iv) C_1 and C_2 are the numbers of clear main effects and clear 2fi's, respectively.

Table S3: 32-run BI-AENP $2^{n-k}:2^r$ designs and comparisons with the MA and CE criteria.

n	k	r	Treatment Add.	Block Add.	BI-AENP ${}^{m}F_{12}$; ${}^{\phi}F_{22}$	WLP $(A_{3,0},\dots,A_{6,0})$ $(A_{2,1},\dots,A_{5,1})$	Cs C_1, C_2
5	0	1		31	$0^5; 0^{10}$	$(0,0,0,0)(0,0,0,1)$	$5,10$
	0	2		7 28	$0^5; 0^{10}$	$(0,0,0,0)(0,2,1,0)$	$5,10$
	0	3		3 14 21	$0^5; 0^8$	$(0,0,0,0)(2,4,1,0)$	$5,8$
	0	4		3 6 12 24	$0^5; -$	$(0,0,0,0)(10,0,5,0)$	$5,0$
6	1	1	31	28	$0^6; 0^{15}$	$(0,0,0,1)(0,2,0,0)$	$6,15$
	1	2	31	7 30	$0^6; 0^{14}$	$(0,0,0,1)(1,4,1,0)$	$6,14$
	1	3	31	6 11 30	$0^6; 0^{12}$	$(0,0,0,1)(3,8,3,0)$	$6,12$
	1	4	31	3 6 12 30	$0^6; -$	$(0,0,0,1)(15,0,15,0)$	$6,0$
7	2	1	15 30	19	$0^7; 0^{15}, 1^6$	$(0,1,2,0)(0,2,2,0)$	$7,15$
	2	2	15 30	14 19	$0^7; 0^{15}, 1^4$	$(0,1,2,0)(2,5,4,0)$	$7,15$
	2	3	15 30	6 12 7	$0^7; 0^{12}, 1^4$	$(0,1,2,0)(5,12,6,2)$	$7,12$
	2	4	14 31	6 12 15 30	$0^7; -$	$(0,2,0,1)(21,0,33,0)$	$7,0$
8	3	1	25 30 15	19	$0^8; 0^{13}, 1^{12}, 2^3$	$(0,3,4,0)(0,3,4,0)$	$8,13$
	3	2	15 25 30	7 17	$0^8; 0^{13}, 1^8$	$(0,3,4,0)(7,2,4,8)$	$8,13$
	3	3	15 25 30	7 17 24	$0^8; 0^{13}$	$(0,3,4,0)(15,6,12,16)$	$8,13$
	3	4	7 14 25	3 6 9 23	$0^8; -$	$(0,5,0,2)(28,0,65,0)$	$8,0$
9	4	1	15 23 27 28	19	$0^9; 0^{15}, -, 2^{18}$	$(0,7,7,0)(3,1,4,4)$	$9,15$
	4	2	15 23 27 28	19 24	$0^9; 0^{15}, -, 2^{12}$	$(0,7,7,0)(9,3,12,12)$	$9,15$
	4	3	15 23 27 28	12 19 24	$0^9; 0^{15}$	$(0,7,7,0)(21,7,28,28)$	$9,15$
	4	4	7 22 25 26	3 17 27 30	$0^9; -$	$(0,9,0,6)(36,0,117,0)$	$9,0$
10	5	1	15 19 21 25 30	17	$0^{10}; -, 1^{40}$	$(0,10,16,0)(5,0,0,16)$	$10,0$
	5	2	15 19 21 25 30	13 17	$0^{10}; -, 1^{36}$	$(0,10,16,0)(9,8,12,32)$	$10,0$
	5	3	15 19 21 25 30	6 13 17	$0^{10}; -, 1^{28}$	$(0,10,16,0)(17,24,36,64)$	$10,0$
	5	4	11 13 21 22 26	15 23 27 30	$0^{10}; -$	$(0,15,0,15)(45,0,195,0)$	$10,0$

(i) Each design is represented by 1, 2, 4, 8, 16 and the numbers specified under "Treatment Add." and "Block Add."

Table S4: 64-run BI-AENP $2^{n-k} : 2^r$ designs and comparisons with the MA and CE criteria.

n	k	r	Treatment Add.	Block Add.	BI-AENP ${}^{m}F_{12}$; ${}^{\phi}F_{22}$	WLP $(A_{3,0},\dots,A_{6,0})$ $(A_{2,1},\dots,A_{5,1})$	Cs C_1, C_2
7	1	1	63	60	$0^7; 0^{21}$	$(0,0,0,0)(0,1,1,0)$	$7,21$
	1	2	47	22 44	$0^7; 0^{21}$	$(0,0,0,1)(0,3,2,1)$	$7,21$
	1	3	63	21 14 60	$0^7; 0^{21}$	$(0,0,0,0)(0,7,7,0)$	$7,21$
	1	4	39	63 21 11 38	$0^7; 0^{16}$	$(0,0,1,0)(5,12,7,3)$	$7,16$
	1	5	47	24 12 6 3 46	$0^7; -$	$(0,0,0,1)(21,0,35,0)$	$7,0$
8	2	1	30 61	27	$0^8; 0^{28}$	$(0,0,2,1)(0,1,2,1)$	$8,28$
	2	2	30 61	23 56	$0^8; 0^{28}$	$(0,0,2,1)(0,4,5,2)$	$8,28$
	2	3	30 61	28 42 27	$0^8; 0^{26}$	$(0,0,2,1)(2,8,10,6)$	$8,26$
	2	4	30 61	49 41 27 60	$0^8; 0^{21}$	$(0,0,2,1)(7,18,15,10)$	$8,21$
	2	5	31 62	12 6 3 30 33	$0^8; -$	$(0,1,0,2)(28,0,69,0)$	$8,0$
9	3	1	15 45 51	21	$0^9; 0^{30}, 1^6$	$(0,1,4,2)(0,1,4,2)$	$9,30$
	3	2	15 45 51	46 26	$0^9; 0^{30}, 1^6$	$(0,1,4,2)(0,6,8,5)$	$9,30$
	3	3	15 45 51	26 13 34	$0^9; 0^{30}$	$(0,1,4,2)(6,10,9,16)$	$9,30$
	3	4	15 45 51	23 56 14 34	$0^9; 0^{24}$	$(0,1,4,2)(12,20,25,36)$	$9,24$
	3	5	49 62 31	6 3 48 15 33	$0^9; -$	$(0,3,0,4)(36,0,123,0)$	$9,0$
10	4	1	42 45 60 51	31	$0^{10}; 0^{33}, 1^{12}$	$(0,2,8,4)(0,2,6,4)$	$10,33$
	4	2	42 45 60 51	27 14	$0^{10}; 0^{33}, 1^{12}$	$(0,2,8,4)(0,8,16,8)$	$10,33$
	4	3	42 45 60 51	39 44 17	$0^{10}; 0^{33}, 1^6$	$(0,2,8,4)(6,16,18,28)$	$10,33$
	4	4	54 49 23 47	28 7 48 38	$0^{10}; 0^{28}$	$(0,3,8,3)(17,24,43,72)$	$10,28$
	4	5	7 14 25 55	45 43 39 23 46	$0^{10}; -$	$(0,7,0,6)(45,0,203,0)$	$10,0$

Table S4: 64-run BI-AENP $2^{n-k}:2^r$ designs and comparisons with the MA and CE criteria (continued).

n	k	r	Treatment Add.	Block Add.	BI-AENP ${}^mF_{12}$; ${}^{\phi}F_{22}$	WLP $(A_{3,0},\ldots,A_{6,0})$ $(A_{2,1},\ldots,A_{5,1})$	Cs C_1, C_2
11	5	1	47 21 57 30 54	19	$0^{11}; 0^{34}, 1^{18}, 2^3$	$(0,4,14,8)(0,2,10,8)$	$11,34$
	5	2	47 21 57 30 54	60 22	$0^{11}; 0^{34}, 1^{18}$	$(0,4,14,8)(3,10,12,20)$	$11,34$
	5	3	47 21 57 30 54	51 22 40	$0^{11}; 0^{34}, 1^{14}$	$(0,4,14,8)(7,22,32,48)$	$11,34$
	5	4	47 21 57 30 54	45 22 39 40	$0^{11}; 0^{28}, 1^4$	$(0,4,14,8)(23,28,74,126)$	$11,28$
	5	5	56 7 59 52 47	5 3 60 15 27	$0^{11}; -$	$(0,11,0,15)(55,0,319,0)$	$11,0$
12	6	1	41 62 35 53 15 27	42	$0^{12}; 0^{36}, 1^{24}, 2^6$	$(0,6,24,16)(0,8,9,0)$	$12,36$
	6	2	41 62 35 53 15 27	11 33	$0^{12}; 0^{36}, 1^{24}$	$(0,6,24,16)(6,16,9,24)$	$12,36$
	6	3	41 62 35 53 15 27	11 58 20	$0^{12}; 0^{36}, 1^{12}, 2^3$	$(0,6,24,16)(15,22,36,96)$	$12,36$
	6	4	41 62 35 53 15 27	23 29 58 20	$0^{12}; 0^{27}, 1^{12}, 2^6$	$(0,6,24,16)(21,54,108,192)$	$12,27$
	6	5	13 11 7 14 25 55	6 12 9 23 46	$0^{12}; -$	$(0,20,0,22)(66,0,475,0)$	$12,0$
13	7	1	52 21 42 37 27 51 57	10	$0^{13}; 0^{36}, -, 2^{39}$	$(0,14,33,16)(3,3,8,24)$	$13,36$
	7	2	52 21 42 37 27 51 57	40 10	$0^{13}; 0^{36}, -, 2^{33}$	$0,14,33,16)(9,9,24,72)$	$13,36$
	7	3	52 21 42 37 27 51 57	49 40 10	$0^{13}; 0^{36}, -, 2^{21}$	$(0,14,33,16)(21,25,52,156)$	$13,36$
	7	4	52 21 42 37 27 51 57	63 15 40 10	$0^{13}; 0^{27}, -, 2^{24}$	$(0,14,33,16)(27,63,156,324)$	$13,27$
	7	5	28 26 7 31 25 14 55	29 24 6 23 57	$0^{13}; -$	$(0,29,0,46)(78,0,686,0)$	$13,0$
14	8	1	52 49 27 37 21 41 13 44	61	$0^{14}; 0^{25}, -^2, 3^{48}, -, 5^{18}$	$(0,39,16,48)(0,16,6,48)$	$14,25$
	8	2	52 49 27 37 21 41 13 44	58 61	$0^{14}; 0^{25}, -^2, 3^{48}, -, 5^{18}$	$(0,39,16,48)(0,28,38,96)$	$14,25$
	8	3	14 22 43 38 62 50 26 44	12 40 54	$0^{14}; 0^{25}, -^2, 3^{32}$	$(0,38,17,52)(34,4,201,99)$	$14,25$
	8	4	14 22 43 38 62 50 26 44	54 40 12 24	$0^{14}; 0^{25}, -^2, 3^{16}, 4^5$	$(0,38,17,52)(66,12,457,203)$	$14,25$
	8	5	56 52 61 55 62 49 14 31	12 9 15 63 17	$0^{14}; -$	$(0,36,0,88)(91,0,965,0,2915)$	$14,0$

Table S4: 64-run BI-AENP $2^{n-k}:2^r$ designs and comparisons with the MA and CE criteria (continued).

n	k	r	Treatment Add.	Block Add.	BI-AENP ${}^{m}F_{12}$; ${}^{\phi}F_{22}$	WLP $(A_{3,0},\ldots,A_{6,0})$ $(A_{2,1},\ldots,A_{5,1})$	Cs C_1, C_2
15	9	1	56 59 11 45 19 35 25 49 42	26	$0^{15}; 0^{27}, -^{3}, 4^{60}, 5^{18}$	$(0,55,22,96)(0,22,6,72)$	$15,27$
	9	2	56 59 11 45 19 35 25 49 42	40 26	$0^{15}; 0^{27}, -^{3}, 4^{60}, 5^{12}$	$(0,55,22,96)(6,45,52,160)$	$15,27$
	9	3	56 59 11 45 19 35 25 49 42	58 40 27	$0^{15}; 0^{27}, -^{3}, 4^{40}$	$(0,55,22,96)(38,5,300,128)$	$15,27$
	9	4	56 59 11 45 19 35 25 49 42	27 40 58 48	$0^{15}; 0^{27}$	$(0,55,22,96)(78,13,660,264)$	$15,27$
	9	5	7 52 13 11 56 62 50 14 28	5 6 12 60 18	$0^{15}; -$	$(0,53,0,136)(105,0,1312,0)$	$15,0$
16	10	1	28 44 50 4 23 38 62 14 26 56	52	$0^{16}; 0^{29}, -^{4}, 5^{84}, 6^{7}$	$(0,77,28,168)(0,28,7,112)$	$16,29$
	10	2	28 44 50 42 23 38 62 14 26 56	20 34	$0^{16}; 0^{29}, -^{4}, 5^{72}$	$(0,77,28,168)(19,2,180,72)$	$16,29$
	10	3	28 44 50 42 23 38 62 14 26 56	48 20 34	$0^{16}; 0^{29}, -^{4}, 5^{48}$	$(0,77,28,168)(43,6,428,160)$	$16,29$
	10	4	28 44 50 42 23 38 62 14 26 56	34 20 48 24	$0^{16}; 0^{29}$	$(0,77,28,168)(91,14,924,336)$	$16,29$
	10	5	28 31 11 26 7 19 14 22 25 47	9 29 24 15 54	$0^{16}; -$	$(0,83,0,230)(120,0,1737,0)$	$16,0$
17	11	1	56 52 44 25 21 51 41 37 61 13 28	17	$0^{17}; 0^{31}, -^{5}, 6^{98}$	$(0,105,35,280)(7,1,84,28)$	$17,31$
	11	2	56 52 44 25 21 51 41 37 61 13 28	48 17	$0^{17}; 0^{31}, -^{5}, 6^{84}$	$(0,105,35,280)(21,3,252,84)$	$17,31$
	11	3	56 52 44 25 21 51 41 37 61 13 28	24 48 17	$0^{17}; 0^{31}, -^{5}, 6^{56}$	$(0,105,35,280)(49,7,588,196)$	$17,31$
	11	4	56 52 44 25 21 51 41 37 61 13 28	17 48 24 12	$0^{17}; 0^{31}$	$(0,105,35,280)(105,15,1260,420)$	$17,31$
	11	5	26 22 13 62 25 21 38 42 50 14 28	27 12 24 60 18	$0^{17}; -$	$(0,92,0,336)(136,0,2288,0)$	$17,0$

(i) Each design is represented by 1, 2, 4, 8, 16, 32 and the numbers specified under "Treatment Add." and "Block Add."
(ii) "$-^{*}$" denotes that the subsequent * elements do not exist.
